# Detecting transmission and reassortment events for influenza A viruses with genotype profile method

**DOI:** 10.1186/1743-422X-8-395

**Published:** 2011-08-09

**Authors:** Changzheng Dong, Liya Ying, Dongfang Yuan

**Affiliations:** 1 School of Medicine, Ningbo University, Ningbo, 315211, China

**Keywords:** influenza A virus, genotype, genotype profile method, reassortment, transmission, phylogenetic analysis, gene segment, IVEE

## Abstract

Evolutionary events of transmission and reassortment for influenza A viruses were traditionally detected by phylogenetic analysis for influenza viruses' eight gene segments. Because the phylogenetic analysis can be complex, we developed genotype profile method which packaged the phylogenetic algorithms to analyze combination patterns of gene segments and integrated epidemiology knowledge. With the method, the analysis of reassortment and transmission becomes a simple and reliable process that combines genotypes, which is identical for the biological process of the virus. An application called IVEE that implements the method is available for all academic users to apply the method http://snptransformer.sourceforge.net. Furthermore, we found that a previous summary of the reassortment events in swine influenza A viruses may be inaccurate.

## Background

Influenza A viruses annually cause seasonal epidemics and occasional global pandemics in humans. Three pandemics occurred in the 20th century, in 1918, 1957 and 1968, which were the result of the transmission of avian viruses or a reassortment between human and avian viruses that greatly changed virus antigenicity [1,2]. At the end of 2008 or the beginning of 2009, a novel swine reassortant was transmitted to humans [3], and a global pandemic broke out in Mexico and USA in April 2009 [4]. Researchers confirmed that the reassorted virus consisted of six gene segments that emerged from triple-reassortant viruses circulating in North American swine and two gene segments from Eurasian avian-like swine H1N1 viruses [3,4]. Because the common ancestor of the new swine-origin influenza A (H1N1) virus (S-OIV) and its most closely related swine viruses existed approximately 10 years ago, the reassortant viruses may have been circulating in pigs for several years before their transmission to humans [3]. Due to the lack of swine surveillance, the details regarding the reassortment event are unclear.

Phylogenetic analysis has been an essential method for research into the molecular evolution of influenza A viruses, especially for cross-host transmission and reassortment). Holmes et al. sequenced 156 complete genomes of human H3N2 influenza A viruses collected between 1999 and 2004 from New York, USA, and phylogenetic analysis revealed that multiple reassortment events had occurred among the co-circulating clades [5]. Nelson et al. showed that segmental reassortment has played an important role in the genomic evolution of H1N1 since 1918 and that intra-subtype reassortment appeared to be an important process in the evolution and epidemiology of H1N1 influenza A virus [6]. Nelson et al. found that multiple clades of both H1N1 and H3N2 entered and co-circulated in the United States during the 2006-2007 influenza season, even in localities that were distant from major metropolitan areas [7]. These data were concordant with other research by the same group concluding that the stochastic processes of viral migration and clade reassortment played a vital role in shaping short-term evolutionary dynamics [8]. Vijaykrishna et al. discovered a novel swine reassortant in Hong Kong containing genes from both 2009 S-OIV and triple-reassortant virus which implied that swine might be a reservoir of reassortment for 2009 S-OIV [9]. Li et al. revealed avian reassortment patterns of highly pathogenic avian influenza (HPAI) H5N1 virus in eastern Asia [10] and the HAPI H5N1 virus had cross-host transmitted to human and caused fatal respiratory illness [11].

**Figure 1:**
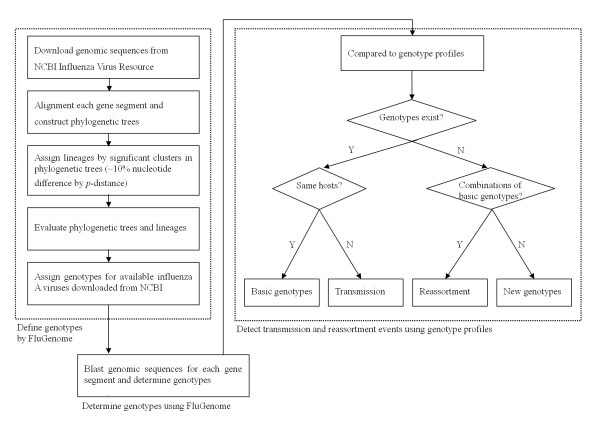
**Workflow of detecting transmission and reassortment events using genotype profile method**. Left part: FluGenome [14] determines genotypes for influenza A viruses. Right part: Candidate genotypes are compared to genotype profiles of human, swine and avian (**Table 1 **Additional file 1, **Table S1**) to test whether they are of following genotypes: basic genotypes, transmission genotypes, reassortment genotypes or novel genotypes. An application called IVEE is freely available to facilitate the detecting process.

Traditionally, transmission and reassortment events have been mostly revealed by separate phylogenetic analysis for the eight gene segments [5-11]. This analysis method is not so straightforward, and the key process lies in identifying the lineages to which each gene segment belongs, a process that requires professional knowledge about numerous virus lineages. Rabadan et al. proposed an interesting method for revealing potential reassortment that calculates the paired nucleotide differences of the third codon positions between the same segments of any two virus strains [12,13]. If the two viruses have a common origin, the differences between all eight segment pairs should be proportional. In contrast, a violation of this rule probably indicates potential reassortment events. The method sounds reasonable; however, several factors may interfere with the calculation of the differences, such as time since divergence, number of generations and geographical isolation. Most importantly, it is difficult to parse the exact parents for potential reassortment. Lu et al. introduced the concept of genotype to define gene segment combinations [14] and developed an online tool called FluGenome [15] to determine genotypes for influenza A viruses and to detect virus reassortment in theory. Lineages for each segment are assigned by a cutoff of 10% nucleotide difference by p-distance in the phylogenetic tree of all nearly complete sequences of influenza A viruses (see Figure 1). The genotypes can be determined by comparing the genomic sequences of new viruses with the genome database using the BLAST algorithm. The best BLAST results are used to assign lineages to the viruses and create genotypes by the sequential combination of the lineages for each segment in the gene order. Thus, the nomenclature of influenza A viruses consists of all eight gene segments, rather than the serotype of hemagglutinin and neuraminidase alone. Reassortment can be detected, in theory, by combining the known genotypes in the database. Unfortunately, FluGenome only provided the process for determining the genotypes, and the analysis process for the reassortment was not implemented directly. Furthermore, the hundreds of genotypes that are collected in the database complicate the analysis even in theory.

In this study, we introduce the concept of "genotype profile" based on "genotype" to describe classic or dominant virus strains. With genotype profiles, the genotypes for the viruses were divided into several basic genotypes and various rare genotypes that may be transmitting viruses or reassortants. Employing genotype profile method, analyzing reassortment and transmission events is a simple and reliable process that combines basic genotypes, similar to the biological process of the virus, while complex phylogenetic analysis is packaged under the method. An application called IVEE that implements the method is available for all academic users to apply the method.

## Methods

### Genotypes

"Genotype" was previously defined by Lu et al. as a sequential combination of the lineages for each of the eight segments in an influenza virus genome [14]. A letter was assigned to each lineage of PB2, PB1, PA, NP and M, and a number followed by a letter was assigned to each lineage of HA, NA and NS, with the number representing the serological subtype or allele. For example, [A, D, B, 3A, A, 2A, B, 1A] is the genotype of human seasonal influenza virus subtype H3N2 with PB2 (1^st ^position) of lineage A, PB1 (2^nd ^position) of lineage D, PA (3^rd ^position) of lineage B, HA (4^th ^position) subtype 3 of lineage A, NP (5^th ^position) of lineage A, NA (6^th ^position) subtype 2 of lineage A, MP (7^th ^position) of lineage B and NS (8^th ^position) subtype 1 of lineage A, which follows the numbering system of influenza genome segments.

We downloaded the genotype information for all influenza A viruses of humans, swine and avian species in the database on 10^th ^Sep, 2010 from FluGenome [15]. In total, there were 3161, 324 and 2572 genomes for human, swine and avian viruses, respectively, and the number of types of corresponding genotypes was 26, 40 and 397, respectively. Two virus strains (A/Texas/09/2009 and A/Canada-ON/RV1527/2009) were used to determine the genotypes of 2009 S-OIV because 2009 S-OIVs and their genotypes were not included in the FluGenome database till now. We downloaded their complete genomic sequences from the NCBI Influenza Virus Resource [16] and determined their genotypes via FluGenome. Most of the lineages of gene segments could be confirmed directly; however, the lineage of NA was assigned using looser parameters (80% coverage and 80% identity).

### Genotype profiles

We defined genotype profiles as lists of all genotypes of classic or predominant virus strains for humans, swine or avian species to filter hundreds of not so important genotypes and import epidemiological knowledge for viruses (see Table 1). With such a definition, the genotypes for the viruses could be divided into common genotypes and rare genotypes. Common genotypes meant that these genotypes occur frequently and mainly refer to classic or predominant virus strains, whereas rare genotypes have low frequencies. However, in some cases, such as human infecting, highly pathogenic avian H5N1 viruses, they should not be considered genotypes in the human genotype profile (we name them basic genotypes) because they have not yet adapted to human hosts, despite the fact that these genotypes were observed in human hosts with high frequencies. Similarly, some reassortants that have not adapted to their hosts may be isolated with high frequency due to frequent sampling. Thus, the difference between common genotypes and basic genotypes depends on whether the hosts are natural hosts or new hosts after adaptation and are judged based on epidemiological knowledge of influenza A viruses. We further assumed that most of the viruses that have rare genotypes are transmitting viruses or reassortants that emerged from combinations of basic genotypes, and would test the hypothesis later. Occasionally some rare genotypes such as [A, A, A, 1A, A, 1A, B, 1A] for A/Brevig Mission/1/1918 had low frequencies due only to the lack of sampling. These genotypes were all excluded from the genotype profiles to decrease the complexity of the genotype profiles. Genotype profiles for human, swine and avian species were established by the following steps: (i) divide genotypes for the viruses into common genotypes and rare genotypes with a cutoff of 5 for genotype frequencies; (ii) common genotypes were further checked based on knowledge of the evolutionary history of influenza A virus to distinguish basic and non-basic genotypes; (iii) basic genotypes in genotype profiles were further classified into groups and subtypes; (iv) rare genotypes and non-basic genotypes were analyzed following the process for detecting transmission and reassortment events described below.

**Table 1:** Genotype profiles for human, swine and avian influenza A viruses

Hosts	Human		Swine			Avian	
**Group**	**Classic human**	**2009 S-OIV**	**Classic swine**	**Eurasian avian-like**	**Triple reassortant**	**Waterfowl**	**Domestic poultry**

Pattern^a^	[A, ?, A/B, 4, A, 6, B, 1A]	[C, D, E, 1A, A, 1F, F, 1A]	[B, A, C, 1A, A, 1B, A, 1A]	[F, G, I, 4, F, 6, F, 1E]	[C, D, E, 4, A, 6, A, 1A]	[C, F, E/H, 4, H, 6, E, 1D/2B]	[K/G, G, D/E, 4, F, 6, F, 1E/1F/2A]

							

H1N1	[A, A, A, 1B, A, 1A, B, 1A]^c^	[C, D, E, 1A, A, 1F, F, 1A]	[B, A, C, 1A, A, 1B, A, 1A]	[F, G, I, 1C, F, 1F, F, 1E]	[C, D, E, 1A, A, 1B, A, 1A]	[C, F, E/H, 1D, H, 1E, E, 1D/2B]	

	[A, A, B, 1B, A, 1A, B, 1A]^c^						

							

H1N2				[F, G, I, 1B, F, 2A, F, 1E]	[C, D, E, 1A/1B, A, 2A, A, 1A]		

							

H2N2	[A, E, B, 2A, A, 2A, B, 1A]						

							

H3N2	[A, D, B, 3A, A, 2A, B, 1A]			[F, G, I, 3A, F, 2A, F, 1E]	[C, D, E, 3A, A, 2A, A, 1A]	[C, F, H, 3C, H, 2D/2G, E, 1D]	

	Swine H3N2^d^						

Other subtypes						[C, F, E/H, 4, H, 6, E, 1D/2B]^e^	[K/G, G, D/E, 4, F, 6, F, 1E/1F/2A]^e^

							
1 (PB2)^b^	A	C	B	F	C	C	K/G

2 (PB1)	A/D/E	D	A	G	D	F	G

3 (PA)	A/B	E	C	I	E	E/H	D/E

4 (HA)	1B/2A/3A	1A	1A	1B/1C/3A	1A/1B/3A		

5 (NP)	A	A	A	F	A	H	F

6 (NA)	1A/2A	1F	1B	1F/2A	1B/2A		

7 (MP)	B	F	A	F	A	E	F

8 (NS)	1A	1A	1A	1E	1A	1D/2B	1E/1F/2A

^a^Pattern: the patterns for the lineages of the gene segments. '?': the pattern in the gene segment is ambiguous; 4/6: similar as the symbol '?' and refers to fourth (HA)/sixth (NA) segment; 'A/B': both A and B are conserved in the gene segment.

^b^1~8: gene segments ordered from PB2 to NS. Characters are conserved lineages in the segments while first three lineages underlined are defined as 'genetic codes'.

^c^[A, A, A, 1B, A, 1A, B, 1A] is the genotype for H1N1 subtype dominated in 1930s and 1940s while [A, A, B, 1B, A, 1A, B, 1A] dominated from 1947, reappeared in 1977 and was present up to date.

^d^The genotype of swine subtype H3N2 is listed here due to its entirely transmitting from human H3N2.

^e^Avian have various different subtypes that are listed in Additional file 1, Table S1.

### Detecting transmission and reassortment events

After the genotypes of the candidate viruses are determined, the genotypes are compared to genotype profiles to detect transmission and reassortment events (see Figure 1). The detecting process includes two steps. First, ascertain whether the genotypes exist in the genotype profiles. If the genotypes of candidate viruses exist in the genotype profiles, the genotypes are basic genotypes as long as the sampling hosts are the same as the hosts of the basic genotype or transmission genotypes when they are different. Thus, the virus is a transmission virus if it has switched hosts from the natural host to the host from which it was sampled. For example, the frequent observation of avian genotypes [K, G, D, 5J, F, 1J, F, 1E] and [G, G, E, 5J, F, 1G, F, 1E] in humans provided evidence that humans were infected with avian H5N1 viruses (see Table 2). Otherwise, the genotypes are considered potential reassortants only if they do not exist in the genotype profiles. Next, observe the characteristics of the lineages of the genotypes and infer the possible reassortment parents by combining the basic genotypes of the genotype profiles. In most cases, the reassortment parents and how they were combined can be easily concluded, except for some novel genotypes that have emerged from unknown origins. We can then test the hypothesis that most of the viruses that have rare genotypes are transmitting viruses or reassortants.

**Table 2:** Transmission and reassortment events of human and swine influenza A viruses

Subtype	Human				Swine			
	**Genotype**	**Events^a^**	**N.^b^**	**Year^c^**	**Genotype**	**Events**	**N**.	**Year**

H1N1	[A, A, A, 1A, A, 1A, B, 1A]	First transmission from avian?[17]	1	1918 (1)	[A, A, B, 1B, A, 1A, B, 1A]	Transmission from human [42]	4	2004-2006

	[A, A, C, 1B, A, 1A, B, 1A]	Unknown^d^	1	1936	[A, A, C, 1A, A, 1A, A, 1A]	Unknown^d^	1	1931 (4)

	[A, A, C, 1A, A, 1B, B, 1A]	Transmission from swine^d^[43]	1	1976 (2)	[A, A, C, 1A, A, 1A, B, 1A]	Unknown^d^	1	1935 (5)

	[A, D, B, 1B, A, 1A, B, 1A]	Reassortant by H3N2 & H1N1	5	1978-1979	[A, G, I, 1C, F, 1F, F, 1E]	Reassortant by EA & human^d^[44]	1	1979

	[B, A, C, 1A, A, 1B, A, 1A]	Transmission from CS	4	1976-1991	[B, D, C, 1A, A, 1B, A, 1A]	Reassortant by TRIG & CS [45]	3	2004

	[C, D, E, 1A, A, 1B, A, 1A]	Transmission from TRIG [46]	1	2005	[C, A, C, 1A, B, 1A, B, 1A]	Unknown^d^	3	1931-1942

	[C, D, E, 1B, A, 1A, A, 1A]	Reassortant by TRIG & H1N1 [37]	3	2009	[C, D, C, 1A, A, 1B, A, 1A]	Reassortant by TRIG & CS [45]	1	2003

	[C, A, C, 1A, B, 1A, B, 1A]	Unknown^d^	2	1982-1987	[C, D, E, 1B, A, 1A, A, 1A]	TRIG & human H1N1 [47]	2	2005

					[C, D, E, 1C, A, 1F, F, 1A]	TRIG & EA [35]	1	2010

					[C, F, E, 1D, H, 1E, E, 2B]	Transmission from avian [48]	1	2002

					[F, G, I, 1A, F, 1F, F, 1E]	Reassortant by EA & CS [49]	1	2000

					[F, G, I, 1A, A, 1B, F, 1E]	Reassortant by EA & CS [3]	2	2001

					[F, G, I, 1C, F, 1F, F, 1A]	Reassortant by EA & TRIG [9]	10	2009

								

H1N2	[A, D, B, 1B, A, 2A, B, 1A]	Reassortant by H3N2 & H1N1 [50,51]	26	2002-2003	[A, D, B, 1B, A, 2A, B, 1A]	Reassortant by H3N2 & human H1N1 [45]	1	2003

					[B, A, C, 1A, A, 2A, A, 1A]	Reassortant by CS & H3N2 [49,52]	4	1980-2004

					[B, D, C, 1B, A, 2A, A, 1A]	Reassortant by TRIG & CS [45]	2	2004

					[C, D, E, 1A, A, 2A, F, 1A]	Reassortant by TRIG & EA [3]	2	2003-2004

					[F, G, I, 1A, A, 2A, F, 1E]	Reassortant by EA & CS [3]	1	2001

					[F, G, I, 1A, F, 2A, F, 1A]	Reassortant by EA & CS [9]	1	2009

								

H2N1	[A, A, B, 2B, A, 1A, B, 1A]	Reassortant by H1N1 & H2N2?^d^	1	1977				

								

H2N3					[C, D, E, 2H, A, 3A, A, 1A]	Reassortant by TRIG & avian [53] H2N3 [53]	2	2006

								

H3N1					[C, D, E, 3A, A, 1B, A, 1A]	Reassortant by TRIG & CS [54,55]	5	2004-2006

								

H3N2	[A, A, A, 3A, A, 2A, B, 1A]	Reassortant by H1N1 & H3N2?^d^	1	1968	[A, D, B, 3A, A, 2A, A, 1A]	Reassortant by H3N2 & CS	1	2002

	[C, D, E, 3A, A, 2A, A, 1A]	Transmission from TRIG [38]	1	2005	[F, G, I, 3A, A, 2A, F, 1A]	Reassortant by EA & CS [56]	1	2004

	[F, G, I, 3A, F, 2A, F, 1E]	Transmission from EA [57]	1	1999				

								

H3N3					[C, F, E, 3C, H, 3A, E, 1D]	Transmission from avian [48]	2	2001

H3N8					[C, I, G, 3F, C, 8B, E, 1D]	Transmission from avian [58]	2	2005-2006

								

H4N6					[C, F, E, 4A, H, 6A, E, 1D]	Transmission from avian [59]	1	1999

								

								

H5N1	[G, G, E, 5J, F, 1G, F, 1E]	Transmission from avian [11,60]	13	1997-1998	[K, G, D, 5J, F, 1J, F, 1E]	Transmission from avian [61]	9	2001-2004

	[K, G, D, 5J, F, 1J, F, 1E]	Transmission from avian [10,62,63]	147	2003-2008				

	[L, G, D, 5J, F, 1J, F, 1E]	Transmission from avian	1	2006 (3)				

								

H5N2					[C, G, E, 5H, A, 2D, A, 2A]	Reassortant by TRIG & avian [64]	1	2008

					[G, G, D, 5H, F, 2D, F, 2A]	Transmission from avian [64]	1	2008

								

H7N3	[C, F, E, 7F, H, 3A, E, 1D]	Transmission from avian [65]	1	2004				

								

H7N7	[G, G, D, 7A, F, 7D, F, 1E]	Transmission from avian [66]	1	2003				

								

H9N2	[G, G, D, 9C, F, 2B, F, 1E]	Transmission from avian [67]	1	2003	[G, G, D, 9F, F, 2D, F, 1E]	Transmission from avian	1	2004

	[G, G, E, 9C, F, 2B, F, 1E]	Transmission from avian [68]	1	1999	[K, G, E, 9C, F, 2B, F, 1E]	Transmission from avian [69]	11	1998-2005

	[G, G, E, 9B, F, 2E, F, 1E]	Transmission from avian [60]	2	1999				

^a^Events: possible transmission or reassortment events and most of them were previously reported. CS: classic swine; EA: Eurasian avian-like swine; TRIG: Triple reassortant swine.

^b^N.: the number of the relevant virus strains in FluGenome.

^c^Year: the isolation year of the relevant virus strains in FluGenome. If multiple strains have been isolated, the first and the last years are listed. The numbers in the round brackets following the years refer to some special virus strains in history: (1) A/Brevig Mission/1/1918; (2) A/New Jersey/1976; (3) A/Indonesia/CDC759/2006; (4) A/swine/1931; (5) A/swine/Ohio/23/1935.

^d^Some events are hard to exactly determined due to lack of full information about these old virus strains.

### Comparing swine reassortment patterns with previous work

Rabadan et al. proposed a distance-based statistical method to analyze the reassortment phenomena and applied the method to human and swine influenza A viruses [13]. We collected the strain names of swine viruses from their paper and retrieved their genotypes from FluGenome. A/swine/Miyazaki/1/2006 was not included in the genotype database of FluGenome, and its genotype was determined manually by collecting its relevant genomic sequences from NCBI. A/swine/North Carolina/35922/98's genotype was not determined because of the lack of its entire genome sequence. The genotypes were further used to detect the potential reassortment events by our genotype profile method as described above.

## Results and Discussion

### Genotype profiles and patterns for human, swine and avian influenza A viruses

#### Human

Genotype profiles for human, swine and avian influenza A viruses are listed in Table 1. Corresponding to the classic or predominant virus strains in history and in recent influenza seasons, there are four basic genotypes for human influenza A viruses: [A, A, A/B, 1B, A, 1A, B, 1A] and [C, D, E, 1A, A, 1F, F, 1A] for subtype H1N1 as well as [A, E, B, 2A, A, 2A, B, 1A] for H2N2 and [A, D, B, 3A, A, 2A, B, 1A] for H3N2. This means that the genotypes of most dominant circulating H3N2 viruses belong to [A, D, B, 3A, A, 2A, B, 1A], with PB2 lineage A, PB1 lineage D, PA lineage B, HA subtype 3 lineage A, NP lineage A, NA subtype 2 lineage A, MP lineage B and NS subtype 1 lineage A. The genotype nomenclature system consists of all eight gene segments, rather than serotypes of hemagglutinin and neuraminidase alone. Thus, the status of the whole virus genome is clearly reflected by the combination of the lineages of eight gene segments.

With the help of the genotypes, we could reconstruct the evolutionary history of the human influenza A virus. In 1918, a global pandemic was caused by the influenza A virus subtype H1N1. Taubenberger et al. isolated and sequenced a virus strain named A/Brevig Mission/1/1918, which had the genotype [A, A, A, 1A, A, 1A, B, 1A] (see Table 2). They proposed that the virus was originally transmitted from avian host and adapted to human before the 1918 pandemic [1,2,17,18] while some researchers argued that the evidence for the avian-origin hypothesis was not enough and it might be the result of a reassortment or a recombination between human and swine viruses [19-22]. It's hard to solve the debate exactly due to the lack of sampling viruses dominating in that era and the factors such as constraining selection and reassortment/recombination complicates phylogenetic analysis [19-24]. Regardless of its origin debate, the H1N1 virus varied quickly and caused the subsequent seasonal epidemics [1,2]. During the 1930s and 1940s (the sampling time of strains in FluGenome was 1933-1947), the genotype [A, A, A, 1B, A, 1A, B, 1A], which had a different HA lineage, dominated. In the next decade (the sampling time of strains in FluGenome was 1948-1957), nearly all circulating strains were [A, A, B, 1B, A, 1A, B, 1A], a strain that reappeared in 1977 and remained up to date. In 1957, three avian gene segments (PB1, HA and NA) replaced the corresponding human-adapted segments of influenza A (H1N1) [1,2], and a novel subtype H2N2 with genotype [A, E, B, 2A, A, 2A, B, 1A] was produced. In 1968, similar events occurred and led to the new H3N2 reassortant with genotype [A, D, B, 3A, A, 2A, B, 1A], where PB1 and HA were derived from avian strains [1,2].

#### Swine

Swine influenza A viruses can be classified into classic swine, Eurasian avian-like swine and triple-reassortant swine viruses. Classic swine influenza viruses have the genotype [B, A, C, 1A, A, 1B, A, 1A] and only exist in the subtype H1N1. Because of divergence over long periods of time, the differences between the genotypes of human and classic swine influenza viruses (i.e., subtype H1N1) are large, and only the lineages for PB1, NP and NS are concordant. Around 1980, avian-like swine influenza A viruses appeared in Europe [25] and then appeared in Asia [26], replacing almost all of the previously predominant classic swine viruses [27,28]. The avian-like viruses have the genotype pattern [F, G, I, 4, F, 6, F, 1E], which is entirely different from the patterns for classic swine or human viruses. Furthermore, some gene segments, such as PB1 (lineage G), NP (lineage F), MP (lineage F) and NS (lineage 1E), are similar to segments that are conserved in avian viruses. It is also notable that the swine viruses that are subtype H3N2 have the same genotype, [A, D, B, 3A, A, 2A, B, 1A], as the human viruses. In fact, the swine genotype was transmitted from humans, and the genome sequences of the human and swine H3N2 subtypes were highly homologous to date [29,30]. In 1998, the swine H3N2 virus reassorted with avian viruses and classic swine viruses to produce the novel triple reassortant with the genotype [C, D, E, 3A, A, 2A, A, 1A] [31,32]. The triple reassortants contain PB1 (lineage D), HA (lineage 3A), NP (lineage A), NA (lineage 2A) and NS (lineage 1A) from human H3N2 viruses; PB2 (lineage C) and PA (lineage E) from avian viruses and MP (lineage A) from classic swine viruses. The reassortants spread to subtypes H1N1 and H1N2 via reassortment with classic swine H1N1 viruses and, occasionally, with human H1N1 viruses [33,34]. In April 2009, the first global pandemic in the 21st century broke out in Mexico and the USA, eventually forcing the WHO to increase the pandemic level to the final alert phase (phase 6). The pandemic was caused by a new reassortant originating from swine with the genotype [C, D, E, 1A, A, 1F, F, 1A]. Most of the gene segments of the 2009 S-OIV emerged from the triple-reassortant viruses (i.e., subtype H1N1), whereas NA (lineage 1F) and MP (lineage F) were derived from a Eurasian avian-like swine virus (i.e., subtype H1N1). This is an entirely new genotype that has not been previously observed, although a very similar genotype, [C, D, E, 1C, A, 1F, F, 1A], was found in a swine virus isolated in China in Jan, 2010 (see Table 2) [35]. The latter was also reassorted from triple-reassortant swine viruses isolated in China in 2006 and Eurasian avian-like swine viruses in China, where the avian-like swine viruses provided HA (lineage 1C), NA (lineage 1F) and MP (lineage F). This further confirmed the inference that 2009 S-OIV was first produced about ten years ago.

#### Avian

Because avian species, including waterfowl and domestic poultry, are natural reservoirs for influenza viruses, genetic diversity is abundant and various genotypes are present in avian hosts, especially in waterfowl. Nevertheless, distinct patterns in the genotypes of avian species were clearly observed: [C, F, E/H, 4, H, 6, E, 1D/2B] for waterfowl and [K/G, G, D/E, 4, F, 6, F, 1E/1F/2A] for domestic poultry (see Table 1 and Additional file 1, **Table S1**). Additional file 1, **Table S1 **shows genotypes and typical hosts for multiple subtypes of waterfowl and domestic poultry. For example, waterfowl such as mallard ducks and green-winged teals mainly have the waterfowl genotype pattern regardless of their serotypes. Interestingly, the domestic poultry genotype was present in the viruses of subtype H5N1 isolated from mallards. This may have been the result of bi-directional virus exchange [10,36]. Similarly, most genotypes of chicken and duck viruses maintain the domestic poultry genotype pattern, whereas some of the viruses have waterfowl genotypes.

#### Conserved lineages and genotype codes

From genotype profiles, various conserved lineages of genotypes could be observed (see Table 1). For example, lineage A for PB2 is conserved in human H1N1 viruses, whereas lineage B is conserved in classic swine, lineage C is conserved in waterfowl and lineage K/G is conserved in domestic poultry. It is not surprising that lineage C for PB2 could be found in triple-reassortant swine viruses and 2009 S-OIV because PB2 of these viruses emerged from avian viruses. We found that lineage H for NP and lineage E/F for MP are both conserved in avian viruses. Thus, if the lineages for the first three gene segments of the viruses are used, we may define "genotype codes" for human, swine and avian viruses. [B, A, C] is the genotype code for classic swine viruses, whereas [F, G, I] is for Eurasian avian-like swine viruses and [C, D, E] is for triple-reassortant swine viruses. The genotype code [A, A/D/E, A/B] for classic human viruses is somewhat complex due to their frequent reassortment. Actually, the dominant viruses of subtypes H1N1 and H3N2 have simple codes: [A, A, B] ([A, A, A] for H1N1 viruses in the 1930s and 1940s) and [A, D, B], respectively. It is not surprising that human 2009 S-OIV has the same [C, D, E] code as the triple-reassortant swine viruses. Similarly, the genotype codes for avian viruses are [C, F, E/H] and [K/G, G, D/E]. Using genotype codes, it becomes easy to classify candidate viruses. If the genotype code for a virus is [C, D, E], it is likely a triple-reassortant swine virus or human derivative like 2009 S-OIV. Similarly, it is obvious that [F, G, I] is Eurasian avian-like swine influenza A virus.

#### Detecting transmission and reassortment events

Table 2 lists the potential transmission and reassortment events of human and swine influenza A viruses that belong to rare genotypes when genotype profiles are constructed. For example, the virus strain A/Brevig Mission/1/1918 (genotype [A, A, A, 1A, A, 1A, B, 1A]) is the oldest influenza virus that has been sequenced and was proposed to be transmitted from avian species [17]. There are many avian genotypes in human and swine viruses, with serotypes varying from H5N1 to H9N2, all of which are the result of direct transmission across hosts. Bastien et al. isolated three strains (genotype [C, D, E, 1B, A, 1A, A, 1A]) in Canada that were the products of genetic reassortment between seasonal H1N1 and triple-reassortant influenza viruses [37]. The genotype of 2009 S-OIV, [C, D, E, 1A, A, 1F, F, 1A] (see Table 1), consists of gene segments that originate from the triple-reassortant virus, subtype H1N1 (genotype [C, D, E, 1A, A, 1B, A, 1A]), and Eurasian avian-like swine virus, subtype H1N1 (genotype [F, G, I, 1C, F, 1F, F, 1E]). Transmission and reassortment events are common for subtype H1N1 swine viruses, probably due to the co-occurrence of classic swine, avian-like swine and triple-reassortant swine viruses and swine is the intermediate host for reassortment. Reassortment between triple-reassortant swine or avian-like swine viruses with classic swine (H1N1) or human H1N1 viruses can all be observed. For avian species, transmission events across hosts also occur. The Avian Influenza Virus Sequencing Project operated by the USDA Agriculture Research Service reported transmission events of classic swine viruses (genotype [B, A, C, 1A, A, 1B, A, 1A]) and triple-reassortant swine viruses (genotype [C, D, E, 3A, A, 2A, A, 1A]), most of which were transmitted from swine to turkeys (data not shown). The latter was also confirmed by Olsen et al. in Canada [38]. Because avian viruses have complex genotypes and abundant genetic diversity, it is difficult to trace transmission and reassortment events among avian species. However, it is still possible to infer potential cases of transmission and reassortment between waterfowl and domestic poultry because their genotype patterns are different.

#### Comparing swine reassortment patterns with previous work

Table 3 lists the reassortment events in swine influenza A viruses detected by Rabadan's method [13] in the left part of the table. Most gene segments have one of the two swine-origin lineages (S1 and S2) except for some lineages being avian (A) or human (H) derived. Due to the mixed lineages of S1 and S2, the gene segments may be the result of reassortment. To our surprise, we found that most of the virus strains belonged to the classic swine, triple-reassortant swine and Eurasian avian-like swine virus groups when the results of the genotype profile method were analyzed. Rabadan's distance-based method was based on such a rule: the nucleotide differences at the third codon position between two segments of two strains should be proportional if the two segments have a common origin. A violation of this rule indicates that the co-occurrence of two segments may be the result of reassortment events. The rule will experience problems when the reference strains and segments for swine viruses are not selected carefully. For example, if a classic swine strain is set as the reference strain, then all triple-reassortant viruses will be classified as reassortants because some segments originate from classic swine viruses, whereas others do not. With regard to Eurasian avian-like swine viruses, which have no shared segments with the classic swine virus, their classification results would be uncertain and dependent on paired nucleotide differences. If the differences are proportional by chance, the viruses would be classified as descendants from the reference strain, or they may be classified as reassortants. For example, two Eurasian avian-like swine viruses (A/swine/Italy/1521/98 and A/swine/Cloppenburg/IDT4777/2005) with the genotype [F, G, I, 1B, F, 2A, F, 1E] are considered to have all classic swine lineages by Rabadan's method, whereas our method regards them as basic genotypes for Eurasian avian-like swine viruses that were possibly reassorted from Eurasian avian-like and triple-reassortant swine viruses [39,40]. Another example, consider A/Swine/Indiana/9K035/99 and A/Swine/Minnesota/55551/00, which were reported to be new triple-reassortant swine viruses by Karasin et al [41]. However, Rabadan assumed that all gene segments except HA had the same lineages as classic swine. In fact, the H1N2 reassortant viruses were derived from traditional H3N2 triple reassortants and H1N1 classic swine viruses. Phylogenetic analysis confirmed the results of genotype profile method (see Additional file 1, **Table S2 and **Additional file 1, **Figure S1**). These examples show that our method is easy to use and accurate for analyzing reassortment process, whereas the distance-based method has difficulty dealing with complex situations, although it does work in certain cases. In our opinion, genotypes of triple-reassortant and Eurasian avian-like swine viruses in genotype profiles can be considered transmission and reassortment events. Thus, genotypes such as [B, A, C, 1A, A, 2A, A, 1A] and [C, D, E, 2H, A, 3A, A, 1A] are true reassortants that can be determined by genotype profiles. However, for H1N1 strains belonging to the classic swine viruses, the genotype profile method could not identify whether they are reassortants because our method has difficulty inferring intra-subtype reassortment events within the same host, as will be discussed below.

**Table 3:** Comparing reassortment patterns in swine influenza A viruses with Rabadan's method

Year	Rabadan's method										Genotype profile method		Ref.^a^
	**Strain**	**Subtype**	**PB2**	**PB1**	**PA**	**HA**	**NP**	**NA**	**MP**	**NS**	**Genotype**	**Classification**	

1976	A/swine/Iowa/1/1976	H1N1	S1	S2	S1	S1	S1	S1	S1	S1	[B, A, C, 1A, A, 1B, A, 1A]	Classic swine	
1976	A/swine/Tennessee/15/1976	H1N1	S1	S2	S1	S1	S1	S1	S1	S1	[B, A, C, 1A, A, 1B, A, 1A]	Classic swine	
1976	A/swine/Tennessee/19/1976	H1N1	S1	S1	S1	S1	S1	S1	S2	S1	[B, A, C, 1A, A, 1B, A, 1A]	Classic swine	
1976	A/swine/Tennessee/23/1976	H1N1	S1	S2	S1	S1	S1	S1	S1	S1	[B, A, C, 1A, A, 1B, A, 1A]	Classic swine	
1977	A/swine/Tennessee/48/1977	H1N1	S1	S1	S2	S1	S1	S1	S2	S1	[B, A, C, 1A, A, 1B, A, 1A]	Classic swine	
1977	A/swine/Tennessee/61/1977	H1N1	S1	S1	S1	S1	S1	S1	S2	S1	[B, A, C, 1A, A, 1B, A, 1A]	Classic swine	
1977	A/swine/Tennessee/62/1977	H1N1	S1	S1	S1	S1	S1	S2	S2	S1	[B, A, C, 1A, A, 1B, A, 1A]	Classic swine	
1977	A/swine/Tennessee/64/1977	H1N1	S1	S2	S1	S1	S1	S1	S1	S1	[B, A, C, 1A, A, 1B, A, 1A]	Classic swine	
1977	A/swine/Tennessee/82/1977	H1N1	S1	S1	S1	S2	S1	S2	S1	S1	[B, A, C, 1A, A, 1B, A, 1A]	Classic swine	
1977	A/swine/Tennessee/96/1977	H1N1	S1	S1	S1	S1	S1	S1	S1	S2	[B, A, C, 1A, A, 1B, A, 1A]	Classic swine	
1979	A/swine/Minnesota/5892-7/1979	H1N1	S1	S1	S1	S1	S2	S1	S1	S1	[B, A, C, 1A, A, 1B, A, 1A]	Classic swine	
1981	A/swine/Ontario/6/1981	H1N1	S1	S1	S1	S1	S2	S1	S1	S1	[B, A, C, 1A, A, 1B, A, 1A]	Classic swine	
1986	A/swine/Iowa/1/1986	H1N1	S1	S1	S2	S2	S1	S1	S1	S1	[B, A, C, 1A, A, 1B, A, 1A]	Classic swine	
1988	A/swine/Wisconsin/1915/1988	H1N1	S1	S1	S1	S1	S2	S1	S1	S1	[B, A, C, 1A, A, 1B, A, 1A]	Classic swine	
2004	A/swine/Korea/CAN01/2004	H1N1	S1	S1	S1	S1	S1	S2	S1	S1	[C, D, E, 1A, A, 1B, A, 1A]	Triple-reassortant swine	[25]
2004	A/swine/Spain/53207/2004	H1N1	S1	S1	S1	S2	S1	S2	S1	S3	[F, G, I, 1C, F, 1F, F, 1E]	Eurasian avian-like swine	
2007	A/swine/Ohio/24366/07	H1N1	S1	S1	S1	S2	S1	S2	S1	S1	[C, D, E, 1A, A, 1B, A, 1A]	Triple-reassortant swine	
1998	A/swine/Italy/1521/98	H1N2	S1	S1	S1	S2	S1	S3	S1	S1	[F, G, I, 1B, F, 2A, F, 1E]	Eurasian avian-like swine	[18]
1999	A/Swine/Indiana/9K035/99	H1N2	S1	S1	S1	S2	S1	S1	S1	S1	[C, D, E, 1A, A, 2A, A, 1A]	Triple-reassortant swine	[36]
2000	A/Swine/Minnesota/55551/00	H1N2	S1	S1	S1	S2	S1	S1	S1	S1	[C, D, E, 1A, A, 2A, A, 1A]	Triple-reassortant swine	[37]
2004	A/swine/Zhejiang/1/2004	H1N2	S1	S1	S1	S1	S1	H	S1	S1	[B, A, C, 1A, A, 2A, A, 1A]	Reassortant by classic & H3N2	[19]
2005	A/swine/Cloppenburg/IDT4777/2005	H1N2	S1	S1	S1	S1	S1	S2	S1	S1	[F, G, I, 1B, F, 2A, F, 1E]	Eurasian avian-like swine	[20]
2006	A/swine/Miyazaki/1/2006	H1N2	S1	S1	S1	S1	S1	S3	S1	S1	[B, A, C, 1A, A, 2A, A, 1A]	Reassortant by classic & H3N2	[21]
2007	A/swine/Shanghai/1/2007	H1N2	S1	S1	S1	S2	S1	S1	S1	S1	[C, D, E, 1A, A, 2A, A, 1A]	Triple-reassortant swine	
1998	A/Swine/Nebraska/209/98	H3N2	A	H	A	H	S	H	S	S	[C, D, E, 3A, A, 2A, A, 1A]	Triple-reassortant swine	[36]
2001	A/swine/Spain/33601/2001	H3N2	S1	S1	S1	S2	S1	S2	S1	S1	[F, G, I, 3A, F, 2A, F, 1E]	Eurasian avian-like swine	
2003	A/swine/North Carolina/2003	H3N2	S	S	S	H1	S	H2	S	S	[C, D, E, 3A, A, 2A, A, 1A]	Triple-reassortant swine	
2007	A/swine/Korea/CY04/2007	H3N2	S1	S1	S1	S1	S1	S1	S1	S2	[C, D, E, 3A, A, 2A, A, 1A]	Triple-reassortant swine	[25]
2007	A/swine/Korea/CY07/2007	H3N2	S1	S1	S2	S2	S1	S1	S1	S1	[C, D, E, 3A, A, 2A, A, 1A]	Triple-reassortant swine	[25]
1998	A/swine/North Carolina/35922/98	H3N2	S	H	S	H	S	H	S	S	Unknown		[16]
2004	A/swine/MI/PU243/04	H3N1	S1	S1	S1	S1	S1	S2	S1	S1	[C, D, E, 3A, A, 1B, A, 1A]	Reassortant by triple & classic	[38]
2006	A/swine/Missouri/2124514/2006	H2N3	S1	S2	A	A	S1	A	S1	S1	[C, D, E, 2H, A, 3A, A, 1A]	Reassortant by triple & avian	[39]
2003	A/swine/Alberta/56626/03	H1N1	S1	S1	S2	S1	S1	S3	S1	S1	[B, A, C, 1A, A, 1B, A, 1A]	Classic swine	[40]
2003	A/swine/Ontario/53518/03	H1N1	S3	S3	S2	S1	S3	S1	S1	S1	[C, D, C, 1A, A, 1B, A, 1A]	Reassortant by triple & classic	[40]
2003	A/swine/Ontario/57561/03	H1N1	S1	S1	S2	S1	S3	S1	S2	S1	[B, A, C, 1A, A, 1B, A, 1A]	Classic swine	[40]
2004	A/swine/Ontario/48235/04	H1N2	S1	H1	S1	H2	S2	H3	S3	S3	[B, D, C, 1B, A, 2A, A, 1A]	Reassortant by triple & classic	[40]
2004	A/swine/Ontario/11112/04	H1N1	S1	H	S1	S1	S2	S1	S1	S1	[B, D, C, 1A, A, 1B, A, 1A]	Reassortant by triple & classic	[40]
2005	A/swine/Alberta/14722/2005	H3N2	S	S	S	S	S	H	S	S	[C, D, E, 3A, A, 2A, A, 1A]	Triple-reassortant swine	[41]

^a^Ref. numbers refer to citations used in Rabadan's paper [14]

The greatest advantage of the genotype profile is that it is a straightforward method following the real viral reassortment process. In fact, the phylogenetic analysis process is hidden under the definition and construction of genotypes [14]. As shown in Figure 1, lineages were defined as significant clusters (about 10% nucleotide difference by p-distance) in phylogenetic trees constructed by all viruses with full genomic sequences. There is no need to reconstruct the phylogenetic trees as long as the shape of the trees has not changed due to epidemiology and no novel lineage needs to be assigned. The determination of genotypes is then performed by finding a position for the virus in the established trees using a BLAST algorithm. The genotype profile method takes advantage of the phylogenetic algorithms' inferring from sequences, integrates epidemiology knowledge into genotype profiles and keeps the epidemiological interfaces for the users to assemble.

**Figure 2:**
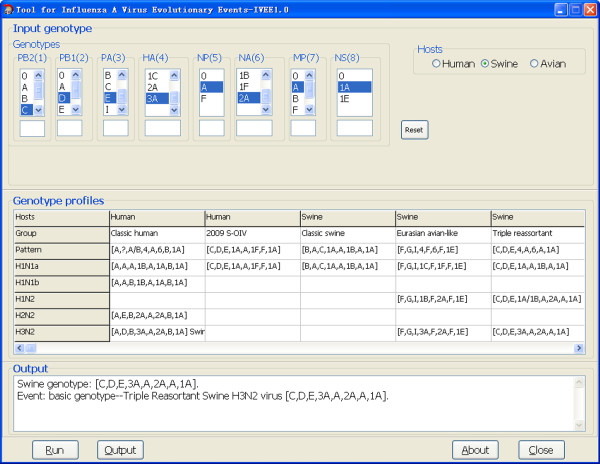
**Screenshot of IVEE**.

An application (see Figure 2) called "Analysis Tool for Influenza A Virus Evolutionary Events (IVEE)" that implements genotype profile method is available for all academic users from the website http://snptransformer.sourceforge.net. In current version, the genotype profiles as shown in Table 1 are embedded and fixed in the program by the authors. The custom genotype profile function will be implemented in the next version, which allows the users to edit the genotype profiles to satisfy various research demands such as updating the newly-discovered genotypes and studying the historic reassortment events. Furthermore, each genotype will be associated with a representative virus strain to help read the analysis results.

The genotype profile based method still had limitations when inferring intra-subtype reassortment within the same host. For example, nearly all human H3N2 viruses have the genotype [A, D, B, 3A, A, 2A, B, 1A], which restrains distinguishing intra-subtype reassortment events as Holmes [5] and Nelson have done [6]. The reason is that a cutoff of a 10% nucleotide difference by p-distance for defining lineages in the phylogenetic tree is too coarse to distinguish some virus clades among subtypes, although it is sufficient for inter-subtype reassortment. One possible solution is to define clades under lineages by FluGenome with lower cutoffs such as 1%. In fact, the WHO/OIE/FAO H5N1 Evolution Working Group has developed a web tool for HPAI H5N1 HA clade prediction http://h5n1.flugenome.org based on FluGenome with average distances of ≧1.5% between clades. The tool and its unified nomenclature system for the HA clade designation of HPAI H5N1 viral strains were used later to facilitate resolution of the nomenclature problem and to make comparisons among virus clades easier across publications. However, the clade designation was designed only for HA without taking into account other gene segments. We suggest that a similar international committee should be established to assign unified clades for all segments of influenza A viruses. This will greatly help research and increase our knowledge of the evolutionary tendency of influenza A viruses.

## Conclusions

In conclusion, we extended the concept of "genotype" to "genotype profile" to describe classic or dominant virus strains and constructed the genotype profiles for influenza A viruses of humans, swine and avian species. Genotype profiles not only decrease the complexity of combinations of hundreds of genotypes but also provide epidemiological information of influenza A viruses for the analysis. With genotype profile method, the analysis of reassortment and transmission events is a simple and reliable process that combines genotypes. We detected various transmission and reassortment events from rare genotypes stored in FluGenome and found that one previous summary of the reassortment events in swine influenza A viruses may be inaccurate. Using genotype profile method, surveillance for virus transmission and reassortment becomes straightforward and it's possible to setup an automatic surveillance system for detecting such evolutionary events.

## Competing interests

The authors declare that they have no competing interests.

## Authors' contributions

CD conceived and designed the study. CD, LY and DY performed the study and drafted the manuscript. All authors read and approved the final manuscript.

## Supplementary Material

Additional file 1 **Table S1**. Genotype profiles for avian influenza A virus. Table S2. The results compared by phylogenetic analysis and genotype profile method. Figure S1. Phylogenetic trees for influenza A virus strains. Click here for file
