# Supplementary material for: Detecting transmission and reassortment events for influenza A viruses with genotype profile method
Source: Virol J. 2011 Aug 9;8:395. doi: 10.1186/1743-422X-8-395 (PMC3162547; doi:10.1186/1743-422X-8-395)
Supplement: Additional file 1 — Table S1. Genotype profiles for avian influenza A virus. Table S2. The results compared by phylogenetic analysis and genotype profile method. Figure S1. Phylogenetic trees for influenza A virus strains. [file 1743-422X-8-395-S1.DOC]

**Table S1. Genotype profiles for avian influenza A viruses**

| Group | Waterfowl | Domestic poultry |
| --- | --- | --- |
| Pattern | [C,F,E/H,4,H,6,E,1D/2B] | [K/G,G,D/E,4,F,6,F,1E/1F/2A] |
| H1N1 | [C,F,E/H,1D,H,1E,E,1D/2B]  mallard (49)^a^, green-winged teal (4) |  |
| H2N1 | [C,F,E,2H,H,1E,E,1D]  mallard (5) |  |
| H2N3 | [C,F,E/H,2H,H,3A,E,1D]  mallard (7) |  |
| H3N2 | [C,F,H,3C,H,2D/2G,E,1D]  mallard (6) |  |
| H3N6 | [C,F,E,3D,H,6A,E,1D]  mallard (2), ruddy turnstone (2) |  |
| H3N8 | [C,F,E/H,3C/3D,H,8A,E,1D/2B]  mallard (27), green-winged teal (13),  pintail (13), northern shoveler (8) | [K,G,D,3B,F,8C,F,2A]  gadwall (3), duck (2) |
| H4N2 | [C,F,E/H,4A,H,2G,E,1D]  mallard (6), blue-winged teal (2) |  |
| H4N6 | [C,F,E/H,4A,H,6A,E,1D/2B]  mallard (42), blue-winged teal (31) |  |
| H4N8 | [C,F,E/H,4A,H,8A,E,1D/2B]  mallard (6), least sandpiper (5) |  |
| H5N1 | [C,F,E,5C,H,1E/2D,E,1D]  mallard(5), shorebird(3) | [K/G,G,D/E,5J,F,1G/1J,F,1E/1F/2A]  chicken (442), duck (303), goose (93),  open-billed stork (28), swan (19),  cygnus olor (18), quail (17), turkey (15),  crow (9), mallard (8), grebe (7) |
| H5N2 | [C,F,E/H,5A/5B/5C,H,2D/2G,E,1D/2B]  duck (23), mallard (9), chicken (13), turkey (5) | [G,G,D,5K,F,2D,F,1E]  poultry (5), chicken (2) |
| H5N3 |  | [K,G,E,5F,F,3B,F,1E]  duck (5) |

^a^The numbers in () are the numbers of relevant virus strains in FluGenome and only typical hosts are listed. Most of them have numbers larger than five while some rare genotypes larger than two.

**Table S1. Continued**

| Group | Waterfowl | Domestic poultry |
| --- | --- | --- |
| Pattern | [C,F,E/H,4,H,6,E,1D/2B] | [K/G,G,D/E,4,F,6,F,1E/1F/2A] |
| H6N1 | [C,F,E,6B,H,1E,E,1D]  northern shoveler (3), American wigeon (2) | [K/G,G,D/E,6B,F,1G/1I,F,1E]  chicken (30), quail (4) |
| H6N2 | [C,F,E,6B/6G,H,2G,E,1D/2B]  mallard (11), pintail (4) | [G,G,E,6A,F,2I,F,1E]  duck (9), wild duck (5) |
| H6N8 | [C,F,E,6G,H,8A,E,1D]  mallard (3) |  |
| H7N1 |  | [G,G,D,7A,F,1H,F,2A]  turkey (28), chicken(6), quail (2) |
| H7N2 | [C,F,E/H,7F,H,2G,E,2B]  chicken (69), guinea fowl (17), turkey (5)  muscovy duck (6), duck (4) |  |
| H7N3 | [C,F,E/H,7F,H,3A,E,1D]  chicken (6), laughing gull (3), mallard (2) | [G,G,D,7A,F,3B,F,1E]  turkey (14), chicken (13) |
| H7N7 |  | [G,G,D,7A,F,7D,F,1E]  chicken (4)、duck (4) |
| H9N2 |  | [K/G,G,D/E,9B/9C/9G,F,2B/2D/2E,F,1E]  chicken (135), turkey (11), duck (4), quail (5) |
| H10N7 | [C,F,E/H,10A,H,7F,E,1D]  ruddy turnstone (12), mallard (11) |  |
| H11N9 | [C,F,E/H,11C,H,9A,E,1D/2B]  mallard (12), green-winged teal (9) |  |
| H12N5 | [C,F,E/H,12A,H,5D,E,1D/2B]  mallard (7), pintail (4) |  |

**Table S2. The results compared by phylogenetic analysis and genotype profile method.**

| Segments | Methods^1^ | A/swine/Italy/1521/98 | A/swine/Cloppenburg/IDT4777/2005 | A/Swine/Indiana/9K035/99 | A/Swine/Minnesota/55551/00 |
| --- | --- | --- | --- | --- | --- |
| PB2 | Tree | Eurasian avian-like | Eurasian avian-like | Eurasian avian-like | Triple reassortant |
|  | Genotype | F | F | C | C |
| PB1 | Tree | Eurasian avian-like | Eurasian avian-like | Triple reassortant | Triple reassortant |
|  | Genotype | G | G | D | D |
| PA | Tree | Eurasian avian-like | ES | Triple reassortant | Triple reassortant |
|  | Genotype | I | I | E | E |
| HA | Tree | Eurasian avian-like ^2^ | Eurasian avian-like ^2^ | Triple reassortant | Triple reassortant |
|  | Genotype | 1B ^2^ | 1B ^2^ | 1A | 1A |
| NP | Tree | Eurasian avian-like | Eurasian avian-like | Triple reassortant | Triple reassortant |
|  | Genotype | F | F | A | A |
| NA | Tree | Eurasian avian-like ^2^ | Eurasian avian-like ^2^ | Triple reassortant | Triple reassortant |
|  | Genotype | 2A ^2^ | 2A ^2^ | 2A | 2A |
| MP | Tree | Eurasian avian-like | Eurasian avian-like | Triple reassortant | Triple reassortant |
|  | Genotype | F | F | A | A |
| NS | Tree | Eurasian avian-like | Eurasian avian-like | Triple reassortant | Triple reassortant |
|  | Genotype | 1E | 1E | 1A | 1A |
| Group report | Both | Eurasian 'avian-like' swine  virus subtype H1N2 | Eurasian 'avian-like' swine virus  subtype H1N2 | Triple reassortant swine  virus subtype H1N2 | Triple reassortant swine virus  subtype H1N2 |

^1^Tree: the result of phylogenetic analysis. Genotype: the result of genotype profile method.

^2^These segments are derived from triple reassortant swine viruses.

**Figure S1. Phylogenetic trees for influenza A virus strains**

Phylogenetic trees were constructed for influenza A virus strains, and four selected virus strains (A/swine/Italy/1521/98, A/swine/Cloppenburg/IDT4777/2005, A/Swine/Indiana/9K035/99, A/Swine/Minnesota/55551/00) were marked with asterisk. Representative virus strains were selected from [1] and [2] , and all sequences for eight genomic segments (PB2, PB1, PA, HA, NP, NA, M1 and NS1) were downloaded from the NCBI Influenza Virus Resource (http://www.ncbi.nlm.nih.gov/genomes/FLU/FLU.html). Maximum likelihood phylogenetic trees were inferred using MEGA 5 under the GTR+I+Γ_4_ (the general time-reversible model with the frequency of each substitution type, the proportion of invariant sites and the gamma distribution of among-site rate variation with four rate categories estimated from the empirical data), which was determined as the best-fit model by MEGA [3]. Phylogeny tests were performed by bootstrap resampling with 100 replicates. The analysis results of four selected virus strains were listed in **Additional file 1, Table S2**.


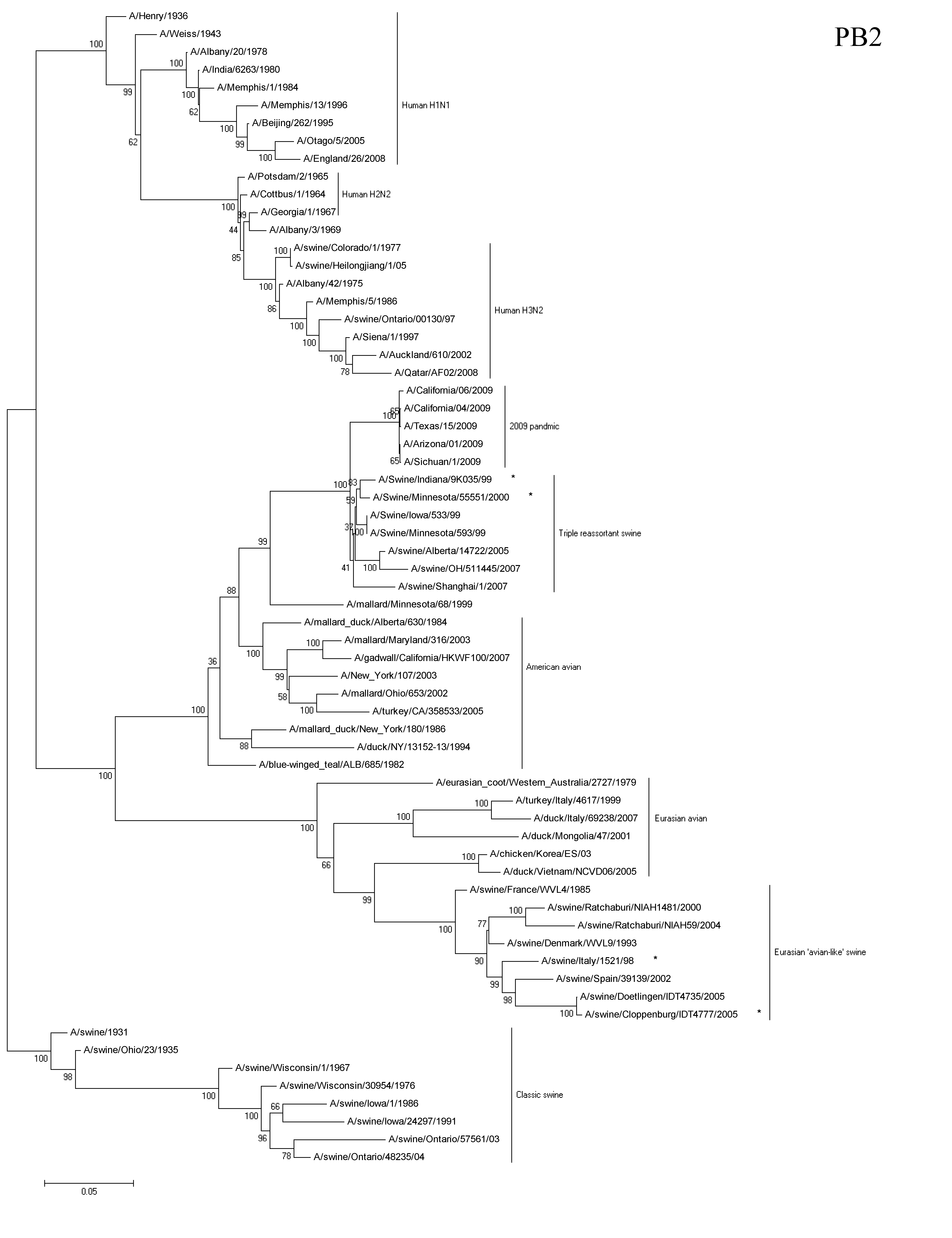


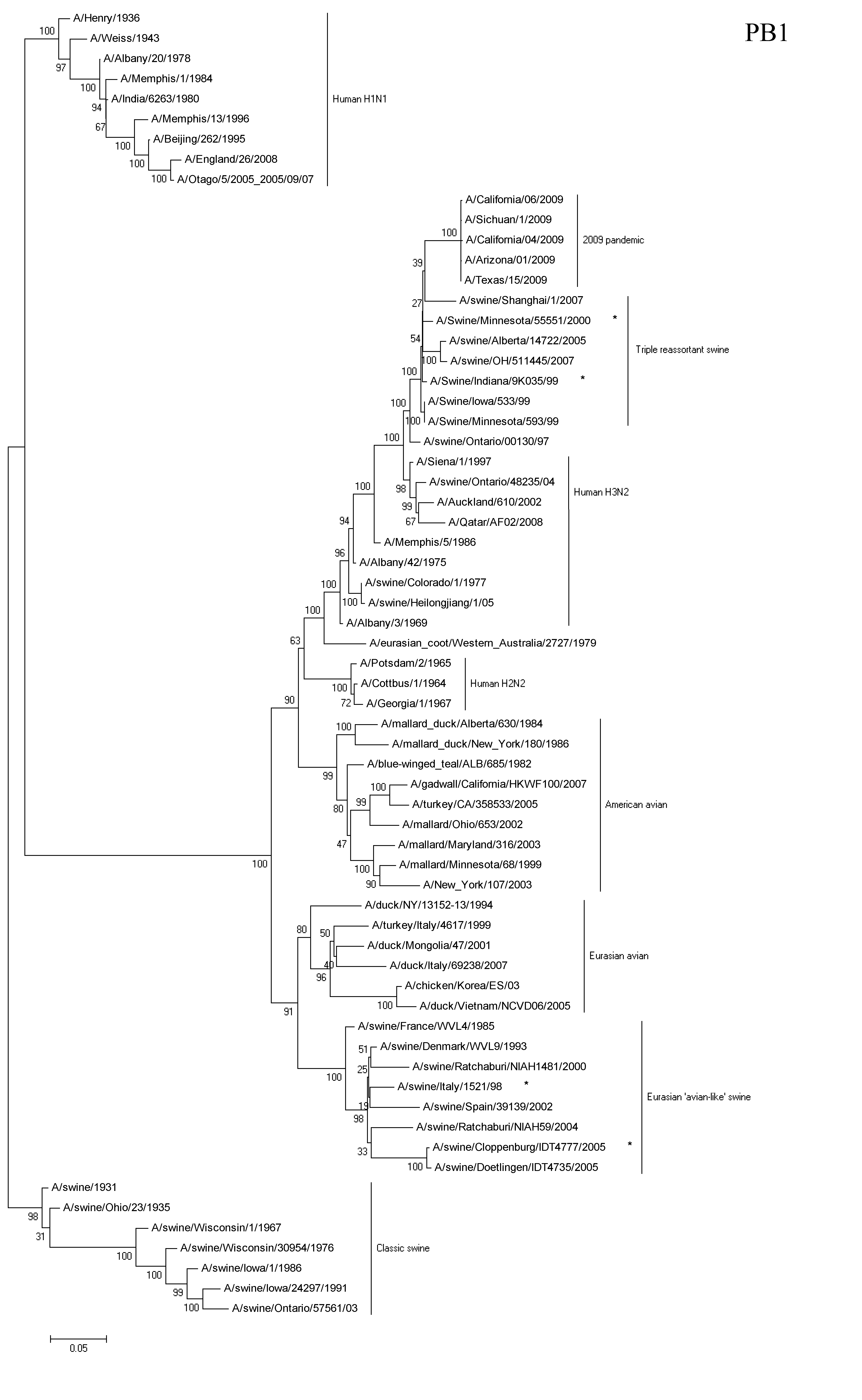


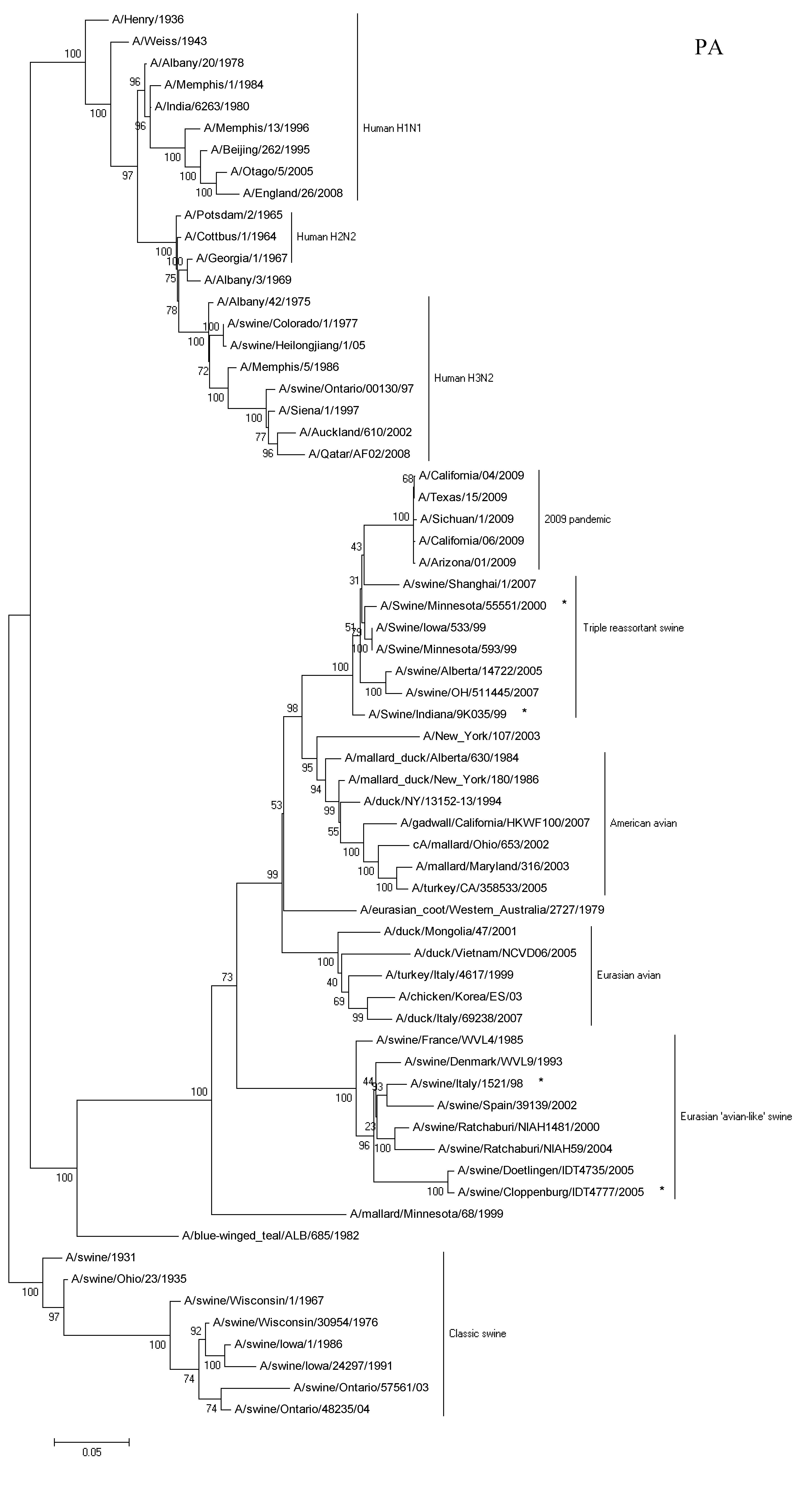


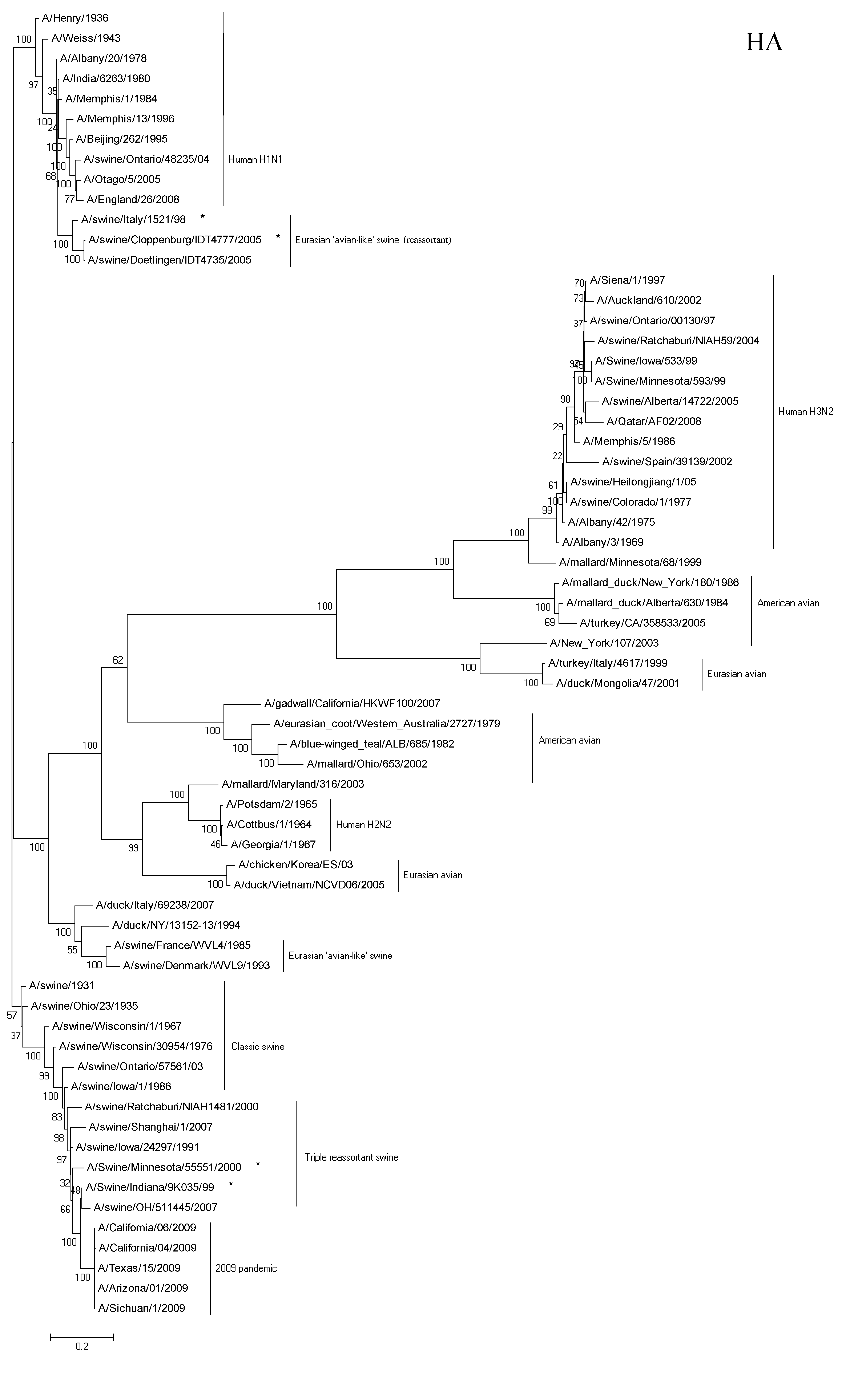


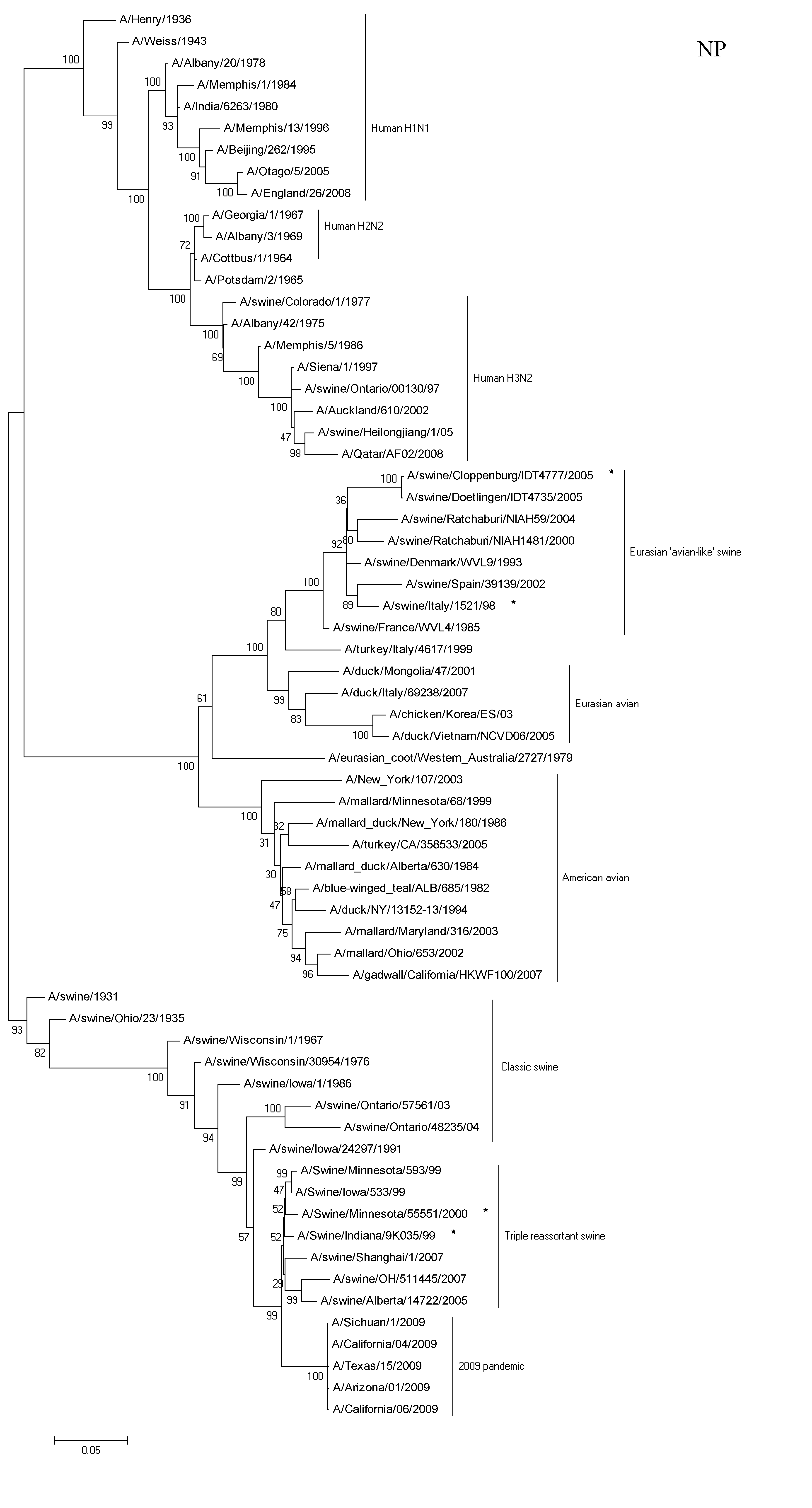


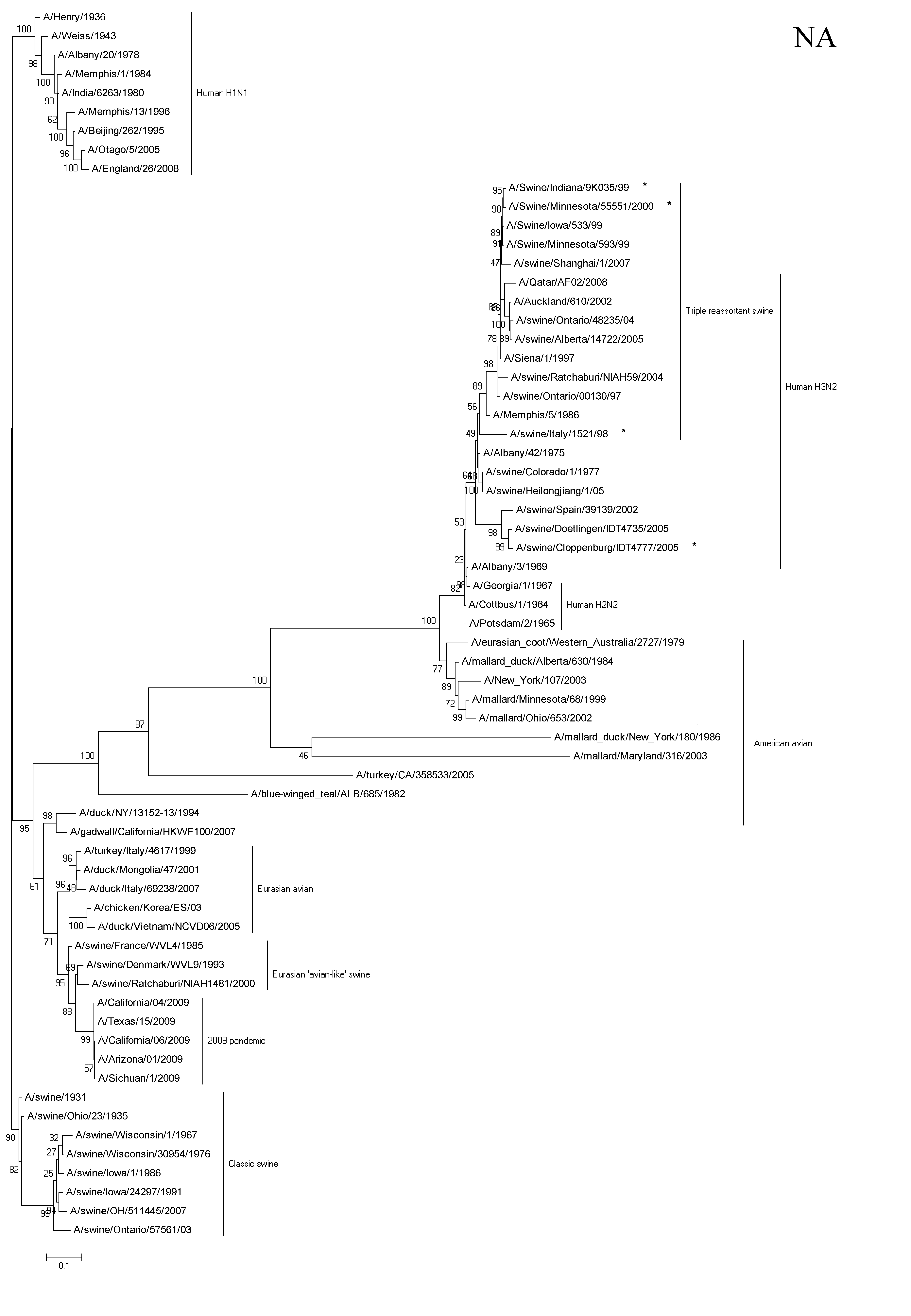


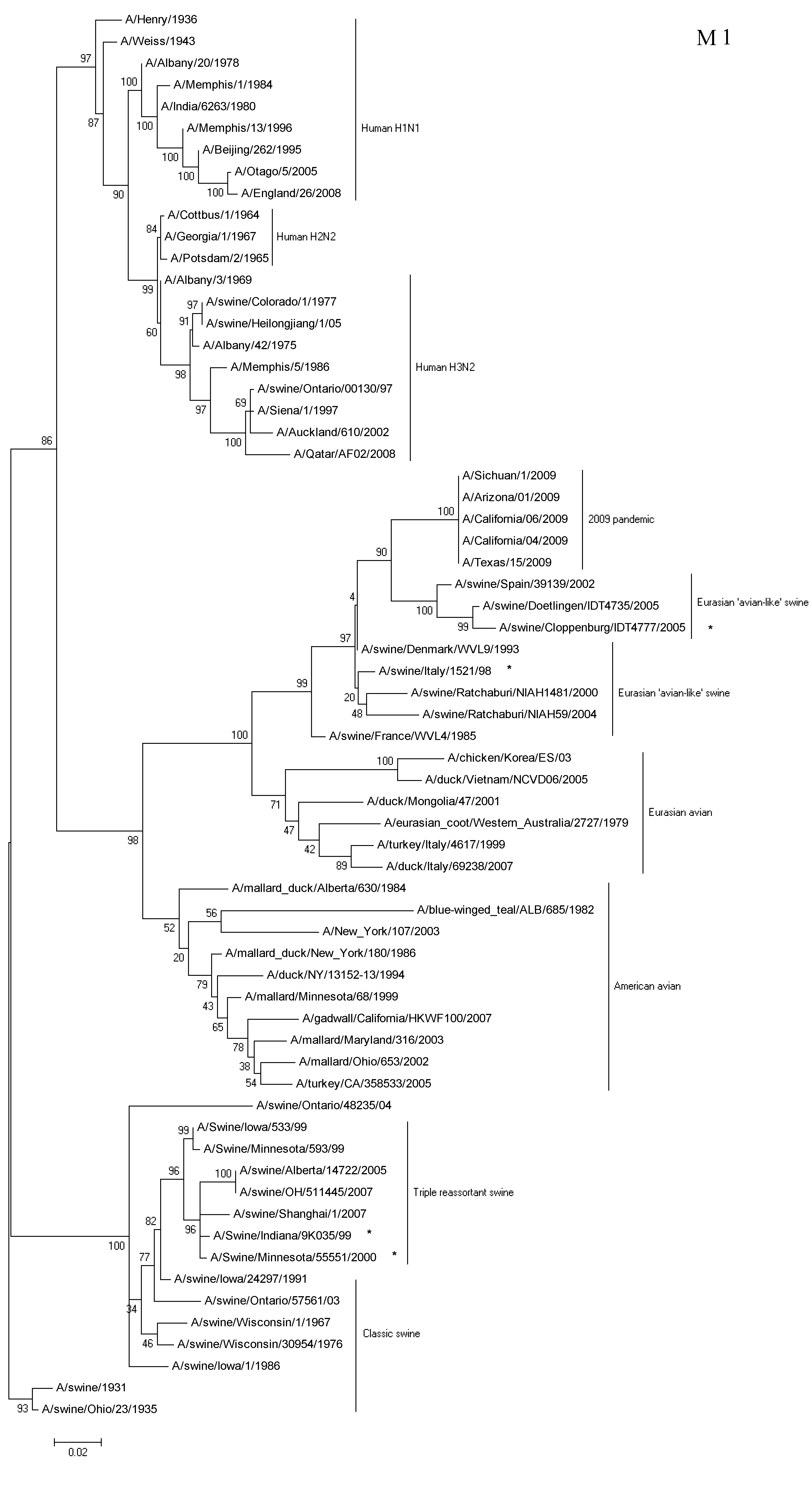


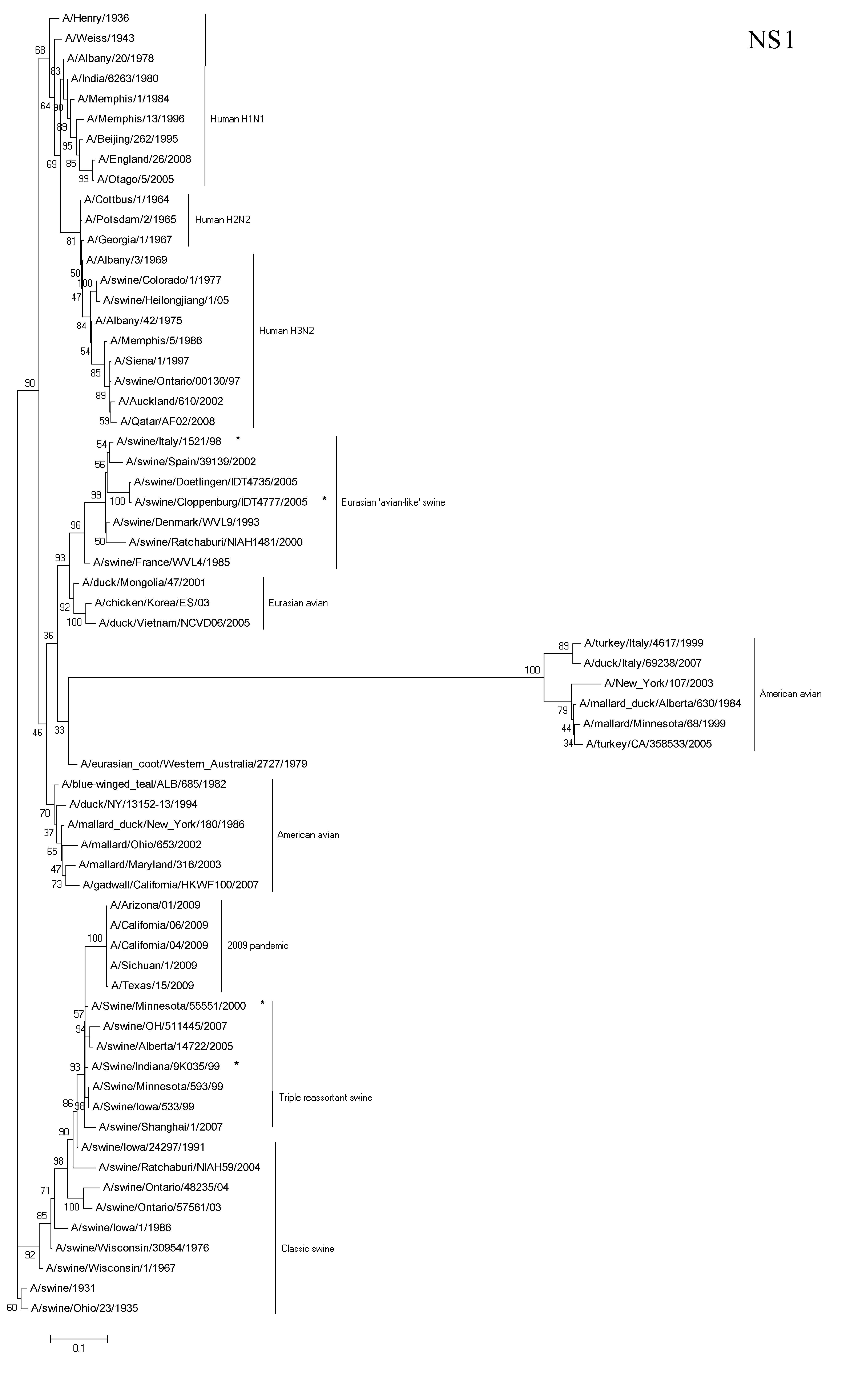


**Additional file 1, references**

1. Garten RJ, Davis CT, Russell CA, Shu B, Lindstrom S, Balish A, Sessions WM, Xu X, Skepner E, Deyde V, et al: **Antigenic and genetic characteristics of swine-origin 2009 A(H1N1) influenza viruses circulating in humans.** *Science* 2009, **325:**197-201.

2. Smith GJD, Vijaykrishna D, Bahl J, Lycett SJ, Worobey M, Pybus OG, Ma SK, Cheung CL, Raghwani J, Bhatt S, et al: **Origins and evolutionary genomics of the 2009 swine-origin H1N1 influenza A epidemic.** *Nature* 2009, **459:**1122-1125.

3. Tamura K, Peterson D, Peterson N, Stecher G, Nei M, Kumar S: **MEGA5: molecular evolutionary genetics analysis using maximum likelihood, evolutionary distance, and maximum parsimony methods.** *Mol Biol Evol*, in press.
